# Effects of Crude *Fucus distichus* Subspecies *evanescens* Fucoidan Extract on Retinal Pigment Epithelium Cells―Implications for Use in Age-Related Macular Degeneration

**DOI:** 10.3390/md17090538

**Published:** 2019-09-16

**Authors:** Kevin Rohwer, Sandesh Neupane, Kaya Saskia Bittkau, Mayra Galarza Pérez, Philipp Dörschmann, Johann Roider, Susanne Alban, Alexa Klettner

**Affiliations:** 1 Department of Ophthalmology, University Medical Center, University of Kiel, 24105 Kiel, Germany; k.rohwer@live.de (K.R.); Philipp.Doerschmann@uksh.de (P.D.); Johann.Roider@uksh.de (J.R.); 2 Department of Pharmaceutical Biology, Pharmaceutical Institute, University of Kiel, 24105 Kiel, Germany; sneupane@pharmazie.uni-kiel.de (S.N.); kbittkau@pharmazie.uni-kiel.de (K.S.B.); mperez@pharmazie.uni-kiel.de (M.G.P.); salban@pharmazie.uni-kiel.de (S.A.)

**Keywords:** *Fucus distichus* subsp. *evanescens*, fucoidan, retinal pigment epithelium, VEGF, oxidative stress, phagocytosis

## Abstract

Fucoidan extracts may have beneficial effects in age-related macular degeneration (AMD). Over-the-counter fucoidan preparations are generally undefined, crude extracts. In this study, we investigated the effect of a crude fucoidan extract from *Fucus distichus* subspecies *evanescens* (Fe) on the retinal pigment epithelium (RPE). Fe extract was investigated for chemical composition and molar mass. It was tested in primary RPE and RPE cell line ARPE19. Oxidative stress was induced with *tert*-butyl hydroperoxide, cell viability evaluated with MTT assay, VEGF secretion assessed in ELISA. Phagocytosis was evaluated in a fluorescence microscopic assay. Wound healing ability was tested in a scratch assay. Additionally, the inhibition of elastase and complement system by Fe extract was studied. The Fe extract contained about 61.9% fucose and high amounts of uronic acids (26.2%). The sulfate content was not as high as expected (6.9%). It was not toxic and not protective against oxidative stress. However, Fe extract was able to reduce VEGF secretion in ARPE19. Phagocytosis was also reduced. Concerning wound healing, a delay could be observed in higher concentrations. While some beneficial effects could be found, it seems to interfere with RPE function, which may reduce its beneficial effects in AMD treatment.

## 1. Introduction

Fucoidans are sulfated polysaccharides derived from brown seaweed, consisting mainly of sulfated fucose. Many different biological activities have been described for fucoidan, but fucoidans are heterogeneous, varying strongly between different species [1]. 

Among the biological activities described for fucoidans are those interesting for potential treatment of age-related macular degeneration (AMD) [2]. AMD is the main cause for blindness and visual impairment in the elderly. Its pathogenesis is complex and multifactorial yet accepted as a major factor in the development of AMD is oxidative stress [3,4,5]. The retina is exposed to high degrees of oxidative stress through constant exposure to high-energetic sun light, due to a high activity of mitochondria in photoreceptors and retinal pigment epithelial cells (RPE), and due to the presence of oxidized fatty acids. The retinal pigment epithelium, a monolayer between the photoreceptors and the choroid, protects the retina from oxidative stress [6] but may succumb to the accumulating damage and degenerate later in life, leading to secondary degeneration of the photoreceptors [4,7,8]. In a subset of AMD, the exudative or wet form, choroidal vessels may grow into the retina, trying to compensate for hypoxia that may be present in the retina due to poor oxygen supply. These vessels are highly immature and leak fluids into the subretinal space, destroying RPE cells and photoreceptors. The most important factor for this neovascularization is vascular endothelial growth factor (VEGF) and VEGF inhibition is the current treatment for exudative AMD [3,9]. 

Fucoidans have been shown to be protective against oxidative stress in various cell assays [10,11,12,13], and we have shown such a protective, anti-oxidative stress effect of fucoidan from *Fucus vesiculosus* in ocular cells as well [14]. Furthermore, a variety of fucoidans have been shown to inhibit VEGF and VEGF-mediated angiogenesis [15,16,17], including in our study on fucoidan of *Fucus vesiculosus* tested on endothelial cells stimulated with RPE supernatant [18]. However, the pro- or anti-angiogenic effect as well as its influence on VEGF are highly dependent on the origin, structure, and molecular weight of the fucoidan [19] and may exert different effects in different experimental systems [14]. 

Most studies have been carried out with commercially available fucoidan from *Fucus vesiculosus*. In this study, we have investigated a fucoidan extract from *Fucus distichus* subspecies *evanescens*. Previous studies on fucoidans from *Fucus evanescens* mainly focused on immunomodulating effects [20,21,22], while there have been only limited studies in the context of potential use for AMD [23]. 

Several studies have reported different structure and composition of fucoidan extracted from *F. evanescens* [20,24,25,26,27]. They described fucose as the main monosaccharide with a low amount of other sugars like mannose, glucose, galactose, and xylose. The diversity in their composition can be dependent on harvest time, place, and the applied extraction method [28].

In our study, we have used a crude extract from *Fucus distichus* subsp. *evanescens* harvested in the Kiel Fjord. The extract was chemically characterized, and some additional basic activities were determined to enable an estimation of its potencies compared to purified fucoidans and was investigated regarding its potential to protect against oxidative stress-induced cell death and to inhibit VEGF secretion. Furthermore, as a functional RPE is a prerogative for functional photoreceptors and needs to be protected to avoid the development of AMD, we additionally tested the effects of the extract on parameters of RPE functions, such as toxicity, phagocytosis, and wound healing. 

## 2. Results

### 2.1. Chemical Characterization of Fe Extract

We determined the basic structural composition of Fe extract (Table 1). Its content of neutral monosaccharides showed to be very low (7.54%), whereas the uronic acid content was quite high (26.1%). The neutral monosaccharides were composed of fucose (61.9%), xylose (10.1%), mannose (24.1%) and glucose (3.9%). Additionally, the molecular weight (Mw) (88.6 ± 1.0 kDa), sulfate content (SO_3_Na; 6.9%), protein content (2.8%), and total phenolic content (TPC; 14.4 ± 0.7 µg GAE/mg) were determined (Table 1).

### 2.2. Activity Assays

Testing of the concentration-dependent inhibitory potency of Fe on elastase and complement system activation revealed half-maximal inhibitory concentrations (IC_50_) of 1.48 ± 0.08 µg/mL (elastase) and 5.73 ± 1.11 µg/mL (complement system) (Table 2). The antioxidant capacity (AOC) of Fe extract (500 µg/mL) amounted to 4.65 ± 1.80%. However, compared to the reference compound Trolox, the effect was about 500 times weaker.

### 2.3. Toxicity of Fe

**Figure 1:**
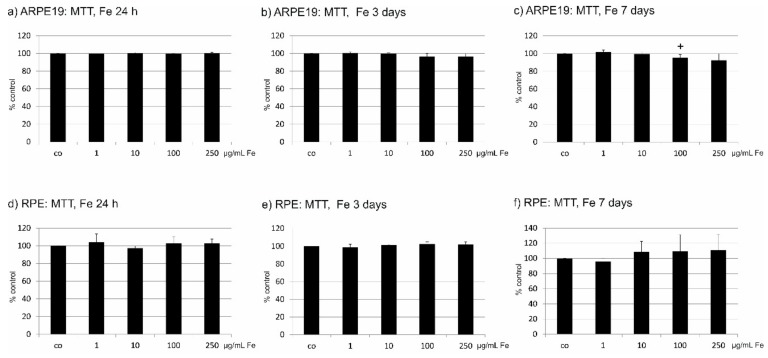
Cell viability tests after incubation with *Fucus distichus* subsp. *evanescens* fucoidan extract for 24 h, three days or seven days. Cell viability was determined by MTT assay. In ARPE19 cells, no influence was found on cells after 24 h (**a**) or three days (**b**). After seven days, a slight but significant reduction of cell viability was seen at a concentration of 100 µg/mL, but not at higher concentrations (**c**). In primary RPE cells, no influence on cell viability was seen after 24 h (**d**), three days (**e**), or seven days (**f**). Significance was evaluated with student’s *t*-test, ^+^ *p* < 0.05, co = untreated control, Fe = crude fucoidan from *Fucus distichus* subsp. *evanescens*, h = hour.

We have tested a potential toxic effect of Fe extract on ARPE19 and primary RPE cells. For ARPE19, no influence of Fe extract in the tested concentrations (1 µg/mL, 10 µg/mL, 100 µg/mL and 250 µg/mL) was found after one day and three days of incubation. After seven days, a slight decrease of cell viability could be noted at a concentration of 100 µg/mL (95.60 ± 3.43%), which reached statistical significance (Figure 1a–c). In primary RPE cells, no influence could be found after 1, 3 or 7 days (Figure 1d–f). In addition, even after four weeks of incubation or after use of 500 µg/mL Fe extract at any tested time point, no loss of cell viability could be seen (data not shown). Consequently, Fe extract does not impair the viability of RPE cells. 

### 2.4. Oxidative Stress Protection

**Figure 2:**
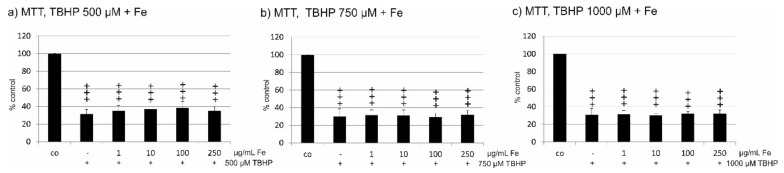
Cell viability after the induction of oxidative stress by *tert*-butylhydroperoxid (TBHP). Cell viability was determined by MTT assay. ARPE19 cells were incubated for 24 h with 500 µM (**a**), 750 µM (**b**), or 1000 µM (**c**) TBHP and the protective effect of Fe extract was measured for 1, 10, 100, and 250 µM. No increase of cell viability was found for any concentration of Fe extract at any oxidative stimulus tested. Significance was evaluated with student’s *t*-test, ^+++^ *p* < 0.001 against untreated control, co = untreated control, Fe = crude fucoidan from *Fucus distichus* subsp. *evanescens.*

Oxidative stress protection has been attributed to fucoidan and to polyphenols, found in crude fucoidan extracts. We tested the protective effect of Fe extract on ARPE19 cells treated with 500, 750, and 1000 µM *tert*-butyl hydroperoxide (TBHP). All three concentrations of TBHP significantly reduced cell viability in ARPE19 cells. When treated with Fe extract (1 µg/mL, 10 µg/mL, 100 µg/mL, and 250 µg/mL), no increase in cell viability was found for any TBHP or Fe extract concentration tested (Figure 2a–c). Clearly, this extract does not provide protection against oxidative stress.

### 2.5. VEGF Secretion

**Figure 3:**
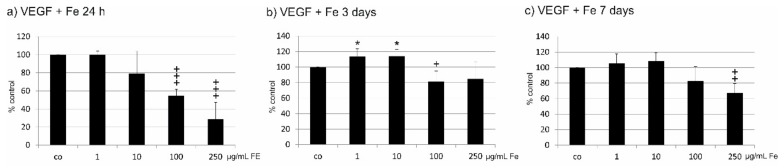
Effect of Fe extract on VEGF secretion of ARPE19 cells. VEGF content in the cell supernatant was investigated with a commercial ELISA. *Fucus distichus* subsp. *evanescens* fucoidan extract was tested in various concentrations (1 µg/mL, 10 µg/mL, 100 µg/mL, 250 µg/mL) for 24 h (**a**), three days (**b**), or seven days (**c**) on ARPE19 cells. After 24 h (**a**), a significant reduction of VEGF could be found for 100 and 250 µg/mL. After three days (**b**), 100 µg/mL was still significantly effective. Of note, a slight but significant increase of VEGF secretion could be found for 1 and 10 µg/mL after three days. After seven days (**c**), 250 µg/mL significantly reduced VEGF content. Significance was evaluated with student’s *t*-test against untreated control, ^+^ *p* < 0.05, ^++^ *p* < 0.01, ^+++^ *p* < 0.001, reduction against untreated control, * *p* < 0.05, increase against untreated control, co = untreated control, Fe = crude fucoidan from *Fucus distichus* subsp. *evanescens*, h = hour.

VEGF secretion was detected in ARPE19 cells after incubation with the different concentrations of Fe extract (1 µg/mL, 10 µg/mL, 100 µg/mL and 250 µg/mL) after 24 h, three days or seven days (Figure 3). At all time points, Fe extract reduced the VEGF concentration in the supernatants compared to untreated control, with the most profound effect after 24 h, which reached statistical significance at concentrations of 100 and 250 µg/mL Fe extract (100 µg/mL: 54.87 ± 7.12%, *p* < 0.001; 250 µg/mL 28.87 ± 18.50%, *p* < 0.001) (Figure 3a). After three days, a significant reduction could be found at a concentration of 100 µg/mL (81.23 ± 13.48%, *p* < 0.05). Of note, 1 and 10 µg/mL resulted in a slight but significant increase of VEGF (1 µg/mL 113.61 ± 9.91%, *p* < 0.05; 10 µg/mL 113.97 ± 9.00%, *p* < 0.05) (Figure 3b). After seven days, a significant decrease of the VEGF content could be found for 250 µg/mL (67.00 ± 12.32, *p* < 0.01) (Figure 3c).

### 2.6. Phagocytosis

**Figure 4:**
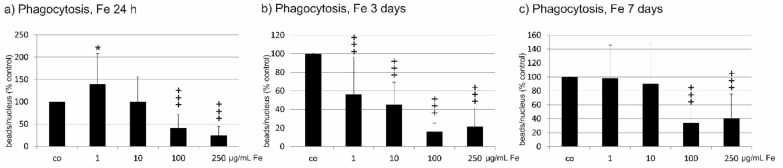
Phagocytic activity of RPE cells after incubation with *Fucus distichus* subsp. *evanescens* fucoidan extract. Phagocytic activity was investigated with a phagocytosis assay using photoreceptor outer segment-treated fluorescent latex beads. RPE cells were treated for 24 h (**a**), three days (**b**), or seven days (**c**) with different concentrations of Fe extract (1 µg/mL, 10 µg/mL, 100 µg/mL, 250 µg/mL). After 24 h, 1 µg/mL induced a significant increase in phagocytic activity, while 100 and 250 µg/mL significantly reduced phagocytosis. After three days, all tested concentrations significantly reduced phagocytosis. After seven days, phagocytosis was significantly reduced by 100 and 250 µg/mL. Significance was evaluated with student’s *t*-test against untreated control, ^+++^ *p* < 0.001, reduction against untreated control, * *p* < 0.05, increase against untreated control. co = untreated control, Fe = crude fucoidan from *Fucus distichus* subsp. *evanescens*, h = hour.

Phagocytosis of shed photoreceptor outer segments is an important task of RPE cells. After incubation with Fe extract for 24 h, 1 µg/mL Fe extract significantly enhanced phagocytic activity (1 µg/mL 139.92 ± 68.32%, *p* < 0.05), while 100 and 250 µg/mL significantly decreased it compared to untreated control (100 µg/mL 41.00 ± 30.75%, *p* < 0.001; 250 µg/mL 24.77 ± 19.94%, *p* < 0.001) (Figure 4a). After three days, all concentrations tested significantly decreased phagocytic activity compared to untreated control (1 µg/mL 56.42 ± 40.34%; 10 µg/mL 45.29 ± 24.05%; 100 µg/mL 16.07 ± 9.39%; 250 µg/mL 21.56 ± 20.02%; all *p* < 0.001) (Figure 4b). After seven days of Fe extract incubation, a significant reduction of phagocytosis compared to untreated cells was seen at 100 µg/mL (33.97 ± 17.35%; *p* < 0.001) and 250 µg/mL (40.82 ± 34.74%; *p* < 0.001) (Figure 4c).

### 2.7. Wound Healing

**Figure 5:**
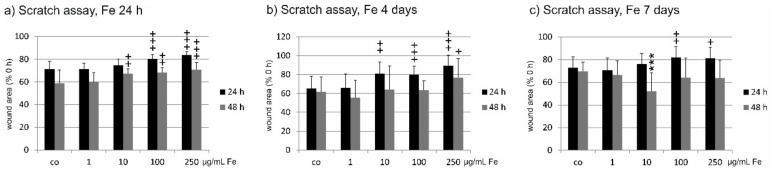
Wound healing of primary RPE cells after incubation with *Fucus distichus* subsp. *evanescens* fucoidan extract. A scratch was applied to a confluent RPE cell layer and the wound area was assessed 24 and 48 h after application. Cells were incubated for 24 h (**a**), four days (**b**), or seven days (**c**) with different concentrations of Fe extract (1 µg/mL, 10 µg/mL, 100 µg/mL, 250 µg/mL). (**a**) When cells were treated for 24 h with Fe extract, wound healing was significantly delayed one day after scratch at 100 and 250 µg/mL. After 48 h, wound healing was significantly delayed at 10, 100 and 250 µg/mL. When cells were treated for four days with Fe extract (**b**), wound healing was significantly delayed one day after scratch at 10, 100, and 250 µg/mL. Forty-eight hours after scratch, a significant delay could be seen at 250 µg/mL. After seven days of Fe extract incubation (**c**) and 24 h after scratch, wound healing was significantly delayed at 100 and 250 µg/mL. This effect was lost 48 h after scratch, where 10 µg/mL significantly accelerated wound healing. Significance was evaluated with student’s *t*-test against untreated control, ^+^ *p* < 0.05, ^++^ *p* < 0.01, ^+++^ *p* < 0.001, delayed wound healing; *** *p* < 0.001, accelerated wound healing, co = scratched control with Fe treatment, Fe = crude fucoidan from *Fucus distichus* subsp. *evanescens*, h = hour.

In the scratch assay, the wound area was analyzed 24 and 48 h post scratch of a confluent cell layer of RPE after treatment for 24 h, four days, or seven days with Fe extract. Incubation with Fe extract for 24 h significantly slowed down wound healing measured 24 h after scratch at 100 and 250 µg/mL Fe extract (control: 71.17 ± 7.16%; 100 µg/mL 80.31 ± 3.67%; 250 µg/mL 83.62 ± 3.18%; (both *p* < 0.001). At 48 h after scratch, also 10 µg/mL as well as 100 µg/mL and 250 µg/mL significantly delayed wound healing (co 58.89 ± 11.54%, 10 µg/mL 67.41 ± 4.30, *p* < 0.01; 100 µg/mL 68.12 ± 4.49, *p* < 0.01; 250 µg/mL 70.81 ± 6.24%, *p* < 0.001) (Figure 5a). After four days of incubation with Fe, wound healing 24 h post scratch was significantly delayed at concentrations of 10 µg/mL (co 65.01 ± 13.34%; 10 µg/mL 80.74 ± 12.42%, *p* < 0.01), 100 µg/mL (79.70 ± 9.03%; *p* < 0.001), and 250 µg/mL (89.05 ± 11.31%; *p* < 0.001) compared to scratched control not treated with Fe extract. But at 48 h after scratch, only 250 mg/mL displayed a significant delay of wound healing (co 61.60 ± 15.69%; 250 µg/mL 76.88 ± 20.12%, *p* < 0.05) (Figure 5b). Long-term incubation with Fe extract for seven days 24 h post-scratch showed a significant delay in wound healing again for 100 µg/mL (co 72.91 ± 9.46%; 100 µg/mL 81.94 ± 9.41%, *p* < 0.01) and 250 µg/mL (81.34 ± 9.71%, *p* < 0.05). After 48 hours, however, this effect was lost and conversely, wound healing was accelerated by 10 µg/mL Fe extract (co 69.54 ± 8.15%; 10 µg/mL 51.93 ± 16.29%, *p* < 0.001) (Figure 5c).

## 3. Discussion

Potential use of fucoidans in medical application has raised much interest [29]. However, the effects of fucoidans may not only differ in dependence on the algae species but also due to the used extraction methods and different degrees of purity [1]. Often, commercially available cosmetics and food supplements are declared to contain fucoidans, but these are generally poorly defined, with considerable deviation in fucoidan content. So far, much research has been done with commercially available fucoidan from *Fucus vesiculosus* [30], including our own study on *Fucus vesiculosus* fucoidan for potential use in AMD or uveal melanoma [14,18]. In the present study, we have investigated a fucoidan from another alga, *Fucus distichus* subsp. *evanescens*, which has so far not received as much attention in the literature. Recently, quite pure fucoidan from *Fucus distichus* subsp. *evanescens* (Fuc-Fe) showed to reduce the VEGF secretion in ARPE19 and displayed high affinity to VEGF but had no protective effect on ARPE19 [23]. In the current study, we used a crude extract of this alga, which can be easily produced in high amounts, elucidating its efficacy.

Despite of the high content of fucose (61%) in the Fe extract, which is the main monosaccharide of fucoidans, the low yield of neutral monosaccharides in the GLC analysis indicate that the content of fucoidan in the Fe extract is quite low. Accordingly, the sulfate content (6.9% as SO_3_Na) was also quite low compared to 15–46% found in crude as well as purified fucoidans from Fucus *distichus* subsp. *evanescens* [20,25,26,27]. This suggests that Fe extract contains far less than 25% fucoidan, whereas the high uronic acid content (26%) indicates a high content of alginic acid, another typical cell wall compound of brown algae. This had to be expected, since methods to remove alginic acid from the extract such as a precipitation with calcium were not applied for the production of Fe extract. As previously shown, the antioxidative capacity of fucoidans is mainly due to co-extracted polyphenols [28,31]. The Fe extract exhibited only weak radical scavenging potency, which was comparable with that of Fuc-Fe and correlated with the respective total phenolic content, which turned out to be lower than that of fucoidan from *Fucus vesiculosus* (manuscript submitted). This is in line with the missing oxidative stress protection of Fe extract (see below).

Regarding a potential use of fucoidan from brown algae as a treatment option for age-related macular degeneration, we tested its effect against oxidative stress, as this comprises a general pathological pathway in AMD, and its interaction with VEGF, as this is the major pathological factor for exudative AMD. 

Fe extract did not exhibit any protection against oxidative-stress induced loss of cell viability in ARPE19 cells. This is in contrast to our finding for fucoidan from *Fucus vesiculosus*, which protected the uveal melanoma from oxidative stress-induced cell death [14], and in correspondence with a paper recently published by our group, which showed a protection by Fuc-Fe of against oxidative stress in uveal melanoma cells but not in ARPE19 [23]. Obviously, different cell types react differently to oxidative stress. Uveal melanoma cell lines are rather susceptible to oxidative stress, as their superoxide dismutase (SOD) activity, which acts in oxidative stress protection, tends to be reduced [32], while RPE are highly resistant to oxidative stress, which is mainly mediated by Nrf-2 [6,33]. Fucoidan has been reported to confer its protection by activation of Nrf-2 and upregulation of SOD [12,13,34], and it is conceivable that this protective pathway may work on one cell line with reduced SOD activity (uveal melanoma) but not with a cell line with constitutive Nrf-2 activation (RPE). However, the lack of any effect concerning oxidative-stress induced cell death strongly indicates that we find no scavenging effect for this Fe extract. These data on oxidative stress protection confirm our previous findings that fucoidan from species other than *Fucus distichus* subsp. *evanescens* may be more suitable for oxidative stress protection [23] but also that the presence of additional compounds in a crude extract does not hold any beneficial effects considering oxidative stress protection.

Concerning VEGF inhibition, Fe extract turned out to reduce VEGF secretion. However, this effect was time- and concentration-dependent, showing the strongest effect after one day. Of note, however, we found an induction of VEGF secretion after three days for lower concentrations of fucoidan (1 and 10 µg/mL), which is not desirable, as the VEGF content in (exudative) AMD eyes has to be reduced. Compared with our data obtained with fucoidan from commercially available *Fucus vesiculosus* and with the purer Fuc-Fe [18,23], our data indicate that Fe extract is less suitable for VEGF secretion inhibition. As this is a crude extract, our data also suggest that no benefit can be seen from additional compounds other than fucoidan present in the extract. Furthermore, fucoidans from other species such as *Saccharina latissima* or *Fucus vesiculosus* may be more promising for further development [23].

RPE cells have a plethora of function in the retina and their functions are vital for a healthy, functioning retina [35]. Furthermore, RPE cells in AMD patients are already challenged and in danger of degeneration. Therefore, any substance to be considered for use in AMD should interfere as little with RPE function as possible. We have tested toxicity, wound healing and phagocytosis as parameters. Similar to our findings with *Fucus vesiculosus* fucoidan as well as fucoidans from five other algae [36], we did not find a relevant toxic effect. However, some minor but nevertheless significant reduction was seen after seven days, which was not observed for *Fucus vesiculosus* fucoidan [18]. Notably, both *Fucus vesiculosus* and Fe extract reduced the wound healing abilities of RPE cells. However, the data obtained 24 and 48 hours after scratch suggest that this is a transient effect and might therefore not be of further consequence for RPE cell function. More importantly, considering the function of RPE cells, Fe extract reduced phagocytic activity of the cells at all tested time points at 100 and 250 µg/mL (and additionally at 1 and 10 µg/mL after three days). A previous study testing fucoidan of *Fucus vesiculosus* at a concentration of 100 µg/mL did not exhibit a reduction of phagocytic activity [18]. In that study, phagocytosis was evaluated only after short term incubation, therefore the effect could be related to duration of fucoidan exposition. However, the effect of the Fe extract was found at every time point tested, indicating a species-dependent effect. In addition, it is possible that other components present in the extract are interfering with phagocytic activity. As a prolonged reduction of phagocytosis could possibly impair the function of the retina, which is not desirable when treating AMD, further testing is needed to elucidate the effects of purity and species of fucoidans on RPE function. 

The results so far indicate some beneficial effects of this crude extract of *Fucus distichus* subsp. *evanescens* with regard to AMD, concerning VEGF inhibition. Previous investigations with the purer Fuc-Fe suggest that beneficial effects are due to the fucoidan content and not due to other compounds of the Fe extract. The reduction of phagocytic activity in RPE cells may be of concern. 

There are other aspects of interest for AMD pathology that we did not test in our assays, such as lipid metabolism [37,38], which may be influenced by fucoidans [39,40], or inflammatory aspects [41], which also could be influenced by fucoidans and especially by fucoidan of *Fucus distichus* subsp. *evanescens* [22,42]. Future studies should address these issues, but for these, highly purified fucoidans should be used. In conclusion, crude extracts from *Fucus distichus* subsp. *evanescens* are of some interests in regard to potential AMD treatment considering their effect on RPE cells. However, fucoidans of other species may be of higher interest, and, importantly, further studies should be performed with highly purified fucoidans. 

## 4. Materials and Methods 

### 4.1. Extraction

*Fucus distichus* subsp. *evanescens* was cleaned from epiphytes and washed with tap water, drained and autoclaved. The material was mixed with four volumes of extraction buffer (100 mM Tris base, pH 10.0) and shredded and blended with Ultraturrax (Sigma-Aldrich, Steinheim, Germany) for 1 min at maximum speed. After centrifugation and separation of the supernatant, the algae material was two further times treated as described with one volume of extraction buffer each. Then, NaCl and citric acid were added to the combined supernatants resulting in 600 mM NaCl and pH 4.75. The extract was then mixed with ethanol (final concentration 50%) (v/v) for precipitation over night at room temperature. After centrifugation, the pellet was dissolved in 20 mM NaOH, and the precipitation procedure including the addition of NaCl and citric acid was repeated once, and was finally dissolved in pure water (pH ~6.0), frozen, and lyophilized. The yield amounted to about 4% in relation to wet algae mass and to 18.4% in dry algae mass. 

### 4.2. Elemental Analysis

The contents of hydrogen, carbon, nitrogen, and sulfur in the crude *Fucus distichus* subsp. *evanescens* fucoidan extract (Fe) were determined by elemental analysis performed with the HEKAtech CHNS Analyser (HEKAtech, Wegberg, Germany; calibrator: sulfanil amide). After gas liquid chromatographic separation (carrier gas: helium), the respective analyte gases were detected in a thermal conductivity detector. The nitrogen content (%) was multiplied by 6.25 to estimate the protein content [43]. Based on the sulfur content (%), the content of sulfate groups (as −SO_3_Na) was calculated. 

### 4.3. Molecular Weight (Mw) Determination

The average molecular weight (Mw) of the fucoidan extract was examined by size exclusion chromatography (SEC) (ÄKTA Pure 25 from GE Healthcare, Munich, Germany), coupled with online multi-angle light scattering (MALS) and refractive index (RI) detection using DAWN 8+ and Optilab T-rex (Wyatt Technology Corporation, Dernbach, Germany). For the separation by hydrodynamic volume, an OHPak LB-806M 8.0 mmID X 300 mmL (ShodexTM, Munich, Germany) column was used. The mobile phase was composed of 0.15 mol/L NaCl, 0.025 mol/L NaH_2_PO_4_, 0.025 mol/L Na_2_HPO_4_ (pH 7.0) and a flow rate of 0.5 mL/min was applied. The sample was dissolved in the elution buffer to a concentration of 2.0 mg/mL, and 100 μL were injected. The elution buffer was degassed using ultrasound for 30 min. The MALS detector was calibrated by the manufacturer using toluol. The used refractive index increment (dn/dc) was 0.150 mL/g. The Mw values were calculated with ASTRA 7.1.2.5 (Wyatt Technology Corporation, Dernbach, Germany). The chromatographic system was controlled by UNICORN 7.2 GE (Healthcare, Munich, Germany).

### 4.4. Monosaccharide Composition by Acetylation Analysis

For the determination of neutral monosaccharide composition, the Fe extract was hydrolyzed with 2.0 mol/L trifluoroacetic acid (TFA) at 121 °C [44] and, after evaporation of TFA, converted into alditol acetate derivatives (AA) by reduction and acetylation [45]. The AA were separated by gas liquid chromatography (GLC) on an OPTIMA-225-0.25 μm fused silica capillary column (25 m × 0.25 mm i.d., film thickness 0.25 μm, Macherey-Nagel, Düren, Germany) using an GC 7890B gas chromatograph (Agilent Technologies, Waldbronn, Germanywith integrated flame ionization detector. The helium flow rate was 1.0 mL/min, the oven temperature was 180 °C for 5 min followed by an increase of 1 °C/min up to 210 °C held for 10 min, the temperatures of injector and detector were 250 °C and 240 °C, respectively. The AA were identified by their retention times. For quantitative analysis, the samples were supplemented with a defined amount of myo-inositol as an internal standard. The percentage of the respective AA was calculated by Agilent MassHunter Qualitative Analysis Workflows B.08.00, (Waldbronn, Germany).

### 4.5. Uronic Acid Determination

Uronic acids were quantified by reaction with 3-hydroxydiphenyl according to the method by Blumenkrantz and Asboe-Hansen modified by Filisetti-Cozzi and Carpita [46].

### 4.6. Total Phenolic Content

The total phenolic content (TPC) was determined by a modified Folin–Ciocalteu method in a microplate format [47] with slightly adapted volumes. Aqueous fucoidan extract (20 μL) was mixed with 0.025 N Folin–Ciocalteu reagent (200 μL; Merck Millipore, Cat. 109001) and incubated for 5 min. Then, 2 M Na_2_CO_3_ (30 μL) was added and absorption was measured at 660 nm (FLUOstar Omega, BMG LABTECH GmbH, Ortenberg, Germany) after 2 h. Gallic acid (Roth, Cat. 7300.1) was used as reference and TPC of sample was expressed as gallic acid equivalents (GAE) in µg per mg of the dry substance.

### 4.7. DPPH Scavenging Assay

The antioxidant potency of the crude *Fucus distichus* subsp. *evanescens* fucoidan extract was determined by the 2,2-diphenyl-1-picrylhydrazyl radical (DPPH; Sigma-Aldrich, Munich, Germany, Cat. D9132) scavenging microplate assay as previously described [48]. An aliquot of 100 µL of a 0.20 mmol/L DPPH-solution in ethanol 70% (V/V) was mixed with 100 µL of the sample (0.5 mg/mL in ethanol 70% (V/V)). For the control, 100 µL DPPH solution were mixed with 100 µL ethanol 70% (V/V). After incubation for 30 min at 20 °C in the dark, the absorption (A) was measured at 520 nm using the plate reader FLUOstar Omega (BMG LABTECH GmbH, Ortenberg, Germany). The radical scavenging potency of the fucoidan samples was calculated by the formula

radical scavenging potency (%) = (A control − A sample)/A control × 100.

Trolox (6-hydroxy-2,5,7,8-tetramethylchroman-2-carboxylic acid; Sigma Aldrich, Munich, Germany) dissolved in ethanol 70% (V/V) was used as reference substance. Its concentration ranged from 3 to 12 µg/mL.

### 4.8. Fluorigenic PMN-Elastase Activity Assay

The elastase inhibitory activity was examined by a fluorogenic microplate assay using elastase from human polymorph nuclear granulocytes (PMN, EC 3.4.21.37, Merck Millipore, Germany) and the substrate MeOSuc-Ala-Ala-Pro-Val-7-amido-4-methylcoumarin (Bachem, Bubendorf, Switzerland) as previously described [49,50]. By means of the concentration-dependent inhibition curves, the concentration of test compound for 50% inhibition of elastase activities (IC_50_ in µg/mL) was calculated.

### 4.9. Hemolytic Classical Complement Modulation Assay

An aliquot of 75 μL fucoidan extract in veronal buffered saline (VBS: 5,5-diethylbarbituric acid 4.94 mmol/L, NaCl 145 mmol/L, MgCl_2_ 0.83 mmol/L, CaCl_2_ 0.25 mmol/L, pH 7.3) was mixed with 50 μL of a hemolytic system consisting of sheep erythrocytes sensitized with rabbit antibodies (Labor Dr. Merk & Kollegen, Ochsenhausen, Germany) in the well of a V-bottom microplate (nunc™ 249570, Thermo Fisher Scientific, Germany). Then, 25 μL of a 2.1% human pooled serum dilution in VBS were added. After incubation for 45 min at 37 °C and subsequent centrifugation for 15 min at 952× *g* at room temperature, 100 μL of the supernatant was transferred into a well of a flat bottom microplate (nunc™ 269620, Thermo Fisher Scientific, Regensburg, Germany) and diluted with 100 μL distilled water. The optical density was measured at 405 nm. For control values, VBS instead of crude Fe extract and hemolytic system were mixed with 2.1% serum dilution (100% hemolysis) and inactivated 2.1% serum dilution (0% hemolysis), respectively. By means of the concentration-dependent hemolysis curves, the IC_50_ (μg/mL) was calculated.

### 4.10. Cell Culture

Primary porcine RPE cells were prepared and cultivated as previously described [51] with modifications [52]. In brief, eyes were obtained from a local slaughterhouse, cleaned, the anterior segment and retina were discarded, and RPE cells harvested by trypsin digestion. Cells were used in the first passage at confluence, morphology, and confluency observed in light microscopy. Cells were maintained in HyClone DMEM (GE Healthcare, Munich, Germany, supplemented with penicillin/streptomycin (1%), HEPES (2.5%), sodium pyruvate (110 mg/mL), and 10% fetal calf serum, Linaris GmbH, Wertheim-Bettingen, Germany). The immortal RPE cell line ARPE19 was obtained from ATCC and cultivated in DMEM (Merck, Darmstadt, Germany), supplemented with penicillin/streptomycin (1%), non-essential amino acids (1%), and 10% fetal calf serum.

### 4.11. Treatment with Fucus distichus subsp. evanescens extract (Fe)

Fe extract was solved in Ampuwa water (Fresenius Kabi, Bad Homburg, Germany) in a concentration of 10 mg/mL and filtered through a 0.2 µm filter. Cells were treated with 1, 10, 100, and 250 µg/mL Fe extract for indicated time periods, diluted in cell culture medium. If stimulation time exceeded three days, medium (including Fe extract) was renewed twice a week.

### 4.12. Oxidative Stress

Oxidative stress was induced by *tert*-butyl hydroperoxide (TBHP), a stable inducer of oxidative stress in RPE cells, as previously described [33]. In this study, we used 500 µM, 750 µM, and 1000 µM TBHP for 24 h on ARPE19 cells. Cells were incubated with indicated concentration of Fe extract for 30 min, then TBHP (500 µM, 750 µM, and 1000 µM, respectively) was added. After incubation for 24 h, cell viability was tested using an MTT test as described below. 

### 4.13. Methyl Thiazolyl Tetrazolium (MTT) Assay

MTT assay is an established viability assay [53] and was conducted as previously described [18]. In brief, cells were incubated with 0.5 mg/mL MTT (3-(4,5-dimethylthiazol-2-yl)-2,5-diphenyltetrazoliumbromid), solved in DMEM without phenol red, washed, and lysed in dimethyl sulfoxide (DMSO). Absorption was measured at 550 nm with a spectrometer (Elx800, BioTek, Bad Friedrichshall, Germany).

### 4.14. VEGF ELISA

ARPE19 supernatants were collected after 24 h, three days and seven days, by quick centrifugation and stored at −20 °C until assessment. VEGF content of the supernatant of ARPE19 cells were determined using a commercially available ELISA kit (R&D Systems), following the manufacturer’s instructions. 

### 4.15. Phagocytosis Assay

Phagocytosis assay was conducted as previously described [54]. In brief, photoreceptor outer segments were prepared from porcine retina and used to opsonize fluorescence-labelled latex beads. Cells were incubated with Fe extract for indicated time periods and treated with opsonized latex beads for four hours. Uptake of beads was detected by fluorescence microscopy (Apotome, Zeiss Microscopy GmbH, Jena, Germany) and evaluated in Axiovision software (Zeiss). 

### 4.16. Wound Healing Assay (Scratch Assay)

Scratch assay was conducted as previously described [18]. In brief, a wound (“scratch”) was applied to a confluent cell layer of primary RPE cells using a pipet tip. Photos were taken immediately after the wound application as well as 24 and 48 h later in light microscopy. Area of wound was assessed with Axiovision software. Wound healing is depicted as % wound area in relation to wound area at time of scratch. 

### 4.17. Statistics

Each experiment was independently repeated at least three times. Calculation of mean, standard deviation, and significance was conducted in Microsoft Excel. Significance was assessed with student’s *t*-test. A *p*-value of 0.05 or below was considered significant. 

## 5. Conclusions

In conclusion, crude extracts from *Fucus distichus* subsp. *evanescens* are of some interests in regard to potential AMD treatment considering their VEGF reducing effect on RPE cells. However, other fucoidans have shown more promising effects. Furthermore, the tested crude extracts interfere with RPE function, such as phagocytosis, which may be a cause of concern. Taken together, fucoidans of other species may be of higher interest, and, importantly, further studies should be performed with highly purified fucoidans. 

## Figures and Tables

**Table 1:** Structural composition of extract from *Fucus distichus* subsp. *evanescens* (Fe).

Monosaccharide Composition (mol %)				Uronic Acid (%)	Mw (kDa)	SO_3_Na (%)	Protein (%)	TPC (µg GAE/mg)
Fuc	Xyl	Man	Glc	26.1 ± 0.2	88.60 ± 1.0	6.9	2. 8	14.4 ± 0.7
61.9	10.1	24.1	3.9					

**Table 2:** Activities of Fe extract.

Elastase Inhibition IC_50_ (µg/mL)	Complement System Inhibition IC_50_ (µg/mL)	DPPH AOC (%) of 500 µg/mL
1.48 ± 0.08	5.73 ± 1.11	4.65 ± 1.80
